# The role of the oral microbiota in chronic non-communicable disease and its relevance to the Indigenous health gap in Australia

**DOI:** 10.1186/s12903-020-01308-y

**Published:** 2020-11-16

**Authors:** Matilda Handsley-Davis, Lisa Jamieson, Kostas Kapellas, Joanne Hedges, Laura S. Weyrich

**Affiliations:** 1 Department of Molecular and Cellular Biology, University of Adelaide, Adelaide, SA Australia; 2 Australian Research Centre for Population Oral Health (ARCPOH), University of Adelaide, Adelaide, SA Australia; 3 Department of Anthropology and Huck Institutes of Life Sciences, The Pennsylvania State University, University Park, PA USA

**Keywords:** Indigenous health, Australia, Microbiota, Microbiome, Oral health, Chronic disease

## Abstract

**Background:**

Aboriginal Australians and Torres Strait Islanders (hereafter respectfully referred to as Indigenous Australians) experience disproportionately poor health and low life expectancy compared to non-Indigenous Australians. Poor oral health is a critical, but understudied, contributor to this health gap. A considerable body of evidence links poor oral health to increased risks of other chronic non-communicable conditions, such as diabetes, cardiovascular disease, chronic kidney disease, and poor emotional wellbeing.

**Main:**

The oral microbiota is indisputably associated with several oral diseases that disproportionately affect Indigenous Australians. Furthermore, a growing literature suggests direct and indirect links between the oral microbiota and systemic chronic non-communicable diseases that underpin much of the Indigenous health gap in Australia. Recent research indicates that oral microbial communities are shaped by a combination of cultural and lifestyle factors and are inherited from caregivers to children. Systematic differences in oral microbiota diversity and composition have been identified between Indigenous and non-Indigenous individuals in Australia and elsewhere, suggesting that microbiota-related diseases may be distinct in Indigenous Australians.

**Conclusion:**

Oral microbiota research involving Indigenous Australians is a promising new area that could benefit Indigenous communities in numerous ways. These potential benefits include: (1) ensuring equity and access for Indigenous Australians in microbiota-related therapies; (2) opportunities for knowledge-sharing and collaborative research between scientists and Indigenous communities; and (3) using knowledge about the oral microbiota and chronic disease to help close the gaps in Indigenous oral and systemic health.

## Background

Aboriginal Australians and Torres Strait Islanders (hereafter respectfully referred to as Indigenous Australians) experience disproportionately poor health and lower life expectancy compared to non-Indigenous Australians. This has been described by the Australian Government as the Indigenous health gap [1]. Gaps in life expectancy and disease burden are largely driven by chronic non-communicable diseases (NCDs). Although many factors contributing to this health gap are well-characterised, there is scant description of the oral microbiota in Indigenous Australians. This is despite growing evidence linking oral microbiota to many oral and systemic chronic NCDs. To contribute to this knowledge gap, we review: (1) current evidence of the extent and causes of the Indigenous health gap in Australia; (2) the oral microbiota, its role in oral and systemic disease, and its influencing factors; and (3) the oral microbiota of Indigenous peoples in Australia and elsewhere. We propose that the oral microbiota is an important, yet under-studied, concept in Indigenous health research and outline both medical and non-medical potential benefits to Indigenous peoples that could result from microbiota research.

### The Indigenous health gap in Australia

Indigenous Australians comprise two distinct First Nations groups in contemporary Australia. Aboriginal Australians are the first peoples of mainland Australia and Tasmania, while Torres Strait Islanders are the first peoples of islands located in the Torres Strait between Australia and Papua New Guinea [2]. The health disparities experienced by Indigenous Australians in comparison to the non-Indigenous population are well-documented. In 2018, the age-standardised death rate for Indigenous Australians was 1.7 times that of non-Indigenous people, while Indigenous Australians had a life expectancy approximately 8 years less than that of their non-Indigenous counterparts [3]. A large proportion of this death and disease burden can be attributed to NCDs such as cancers, diabetes, cardiovascular disease, ischaemic heart disease, chronic respiratory diseases, and poor mental health [4, 5]. Furthermore, several chronic NCDs exhibit both a higher incidence and higher death rate in Indigenous Australians compared to the non-Indigenous population. For example, Indigenous Australians have an incidence of diabetes 3.5 times higher, and a death rate from diabetes approximately 5 times higher, than non-Indigenous Australians. The incidence and death rate for Indigenous Australians from chronic kidney disease are 3.5 times and 2.6 times higher, respectively, than those of non-Indigenous Australians [4]. Addressing the burden of chronic NCDs is therefore a priority in closing the Indigenous health gap in Australia.

Although less widely documented, poor oral health is another chronic NCD prevalent among Indigenous Australians. Unfortunately, representative nationwide data on the oral health status of Indigenous Australians is limited. The 2017–18 National Survey of Adult Oral Health (NSAOH) reported that only 1.7% of participants who underwent dental examinations identified as Indigenous, compared to 2.4% of the Australian population in the 2016 census [6]. Due to this small sample size and lack of representativeness, it is difficult to draw firm conclusions about the oral health of Indigenous Australians from this survey, highlighting the need for more effective approaches to data collection on Indigenous Australian oral health.

Nevertheless, available evidence supports the existence of a substantial oral health gap within the overall Indigenous health gap, with Indigenous Australians experiencing a high incidence and severity of oral diseases including dental caries (tooth decay), periodontal (gum) disease, and head and neck cancers [4, 7–12]. An earlier NSAOH conducted in 2004–2006 reported an Indigenous participation rate similar to that identified in the 2001 census [13]. The 2004–2006 NSAOH found that Indigenous Australians had “disproportionately elevated rates of tooth loss, untreated decay and tooth wear” and experienced worse oral health overall than non-Indigenous counterparts [7]. Specifically, 57% of Indigenous participants in the 2004–2006 NSAOH experienced untreated dental caries, more than twice the proportion of non-Indigenous participants. The 2004–2006 NSAOH additionally found that Indigenous Australians experienced a high prevalence of moderate or severe periodontal disease compared to non-Indigenous counterparts [7]. A smaller convenience study of young adults in an Aboriginal birth cohort in the Northern Territory found a prevalence of untreated tooth decay 3.1 times higher, and of moderate or severe periodontal diseases 10.8 times higher, compared to age-matched NSAOH participants [8]. Research with otherwise healthy Aboriginal Australian adults in the Northern Territory found that 88% had moderate or severe periodontitis, equivalent to 3.5 times the estimated national average [9]. This suggests that the prevalence and severity of periodontal disease among Indigenous Australians may be much higher than what has been captured in nationwide surveys that do not specifically seek out Indigenous participants.

Indigenous Australians also experience a high burden of oral cavity and oropharyngeal cancers [10]. A recent review found Indigenous Australians are diagnosed with head and neck cancers at twice the rate of non-Indigenous Australians [4]. As of 2011, oral cavity and oropharyngeal cancers were responsible for 7% of the total cancer burden for Indigenous Australians living in the state of Queensland, sharing third place with colorectal cancers [11]. Data from the state of South Australia indicate that head and neck cancers represent approximately 8% of cancer diagnoses in Aboriginal South Australians, compared to only approximately 2% of cancer diagnoses in non-Indigenous South Australians [12]. Furthermore, Aboriginal South Australians were more likely to be diagnosed with cancer at a later stage and had a significantly lower 5-year survival rate from cancer compared to non-Indigenous counterparts [12]. Reflecting these state-level findings, cancer has recently overtaken cardiovascular diseases as the leading cause of Indigenous deaths nationwide [3]. Therefore, despite gaps in the data, an overall picture emerges of considerably poorer oral health in Indigenous Australians in comparison to the overall Australian population.

Oral health is a particularly important, yet understudied, contributor to the Indigenous health gap due to the numerous links between oral and systemic health. Periodontal disease has been identified as a risk factor for many chronic NCDs, including diabetes [14–17], chronic kidney disease [18], cardiovascular disease [19, 20], rheumatoid arthritis [21–24], and head and neck cancers [25, 26]. The mouth can also act as a reservoir for bacteria that can cause dangerous infections elsewhere in the body, such as infective endocarditis [27, 28]. Furthermore, poor oral health frequently has negative impacts on mental wellbeing and quality of life. For example, in a recent survey of urban Indigenous Australian adults seeking primary healthcare, approximately 40% of women and 25% of men reported recently experiencing oral pain or discomfort [29]. Similar proportions reported recently experiencing discomfort when eating because of oral health problems, while approximately 24% of women and 14% of men reported recent sleep disruption due to pain from oral health problems [29]. Poor oral health has been associated with anxiety, depression, and suicidal thoughts in young Aboriginal Australian adults [30]. Therefore, improving oral health could lead to a lowered risk of chronic disease and infection as well as improved social and emotional wellbeing and overall quality of life for Indigenous Australians.

These observed disparities in both oral and overall health result from a complex mixture of factors. It is well established that people living in rural and remote areas of Australia often have poorer health outcomes than their urban counterparts. This is due, in part, to these areas having less health infrastructure and a smaller health workforce, particularly of specialist medical professionals [31]. A larger proportion of the Indigenous Australian population lives in outer regional or remote areas (39% of the Indigenous population vs 9.5% of the non-Indigenous population), which may contribute to health disparities between Indigenous and non-Indigenous Australians [32]. However, while many Indigenous Australians experience socioeconomic disadvantage or health challenges related to geographical remoteness, these factors do not fully explain the health gap. While Indigenous Australians are more likely than non-Indigenous Australians to live in rural and remote areas, the majority (60.1%) reside in metropolitan locations or inner regional areas [32]. A study of public dental patients in Australia found that Indigenous Australians experienced noteworthy disparities in oral health even among an economically disadvantaged cohort [33]. Important physical risk factors for poor oral and overall health, such as overweight and obesity, tobacco smoking, and high levels of free sugar consumption, are comparatively prevalent among Indigenous Australians [4, 34]. Socioeconomic and cultural factors, including education and employment, the economic and social costs of obtaining healthcare, and racism and lack of cultural competence in the healthcare system, can prevent Indigenous Australians from seeking and receiving timely and appropriate care [7, 35–37]. Mistrust of the Australian government, unemployment, poor housing, high alcohol and sugar consumption, poor communication of health information, and lack of cultural and linguistic sensitivity in the Australian healthcare system have all been identified by Indigenous individuals and healthcare workers as factors contributing to the health burdens of Indigenous Australians [38–40].

Despite a range of initiatives attempting to address identified contributing factors, progress in closing the health gap to date has been limited [3, 36, 41, 42]. The Council of Australian Governments (COAG) first committed at the end of 2007 to an intention to close the gap in Indigenous life expectancy by 2030 [43]. Specific targets towards this goal, including closing the gap in life expectancy by 2031 and halving gaps in child mortality, literacy and numeracy skills, and employment by 2018, were set by COAG in 2008 [43]. In 2013, several new initiatives were announced by the Australian Government, including the National Aboriginal and Torres Strait Islander Health Plan 2013–2023, a renewed national partnership agreement on closing the gap in Indigenous health outcomes, and an implementation plan for the National Aboriginal and Torres Strait Islander health plan [43]. However, an independent 10-year review of COAG’s Closing the Gap (CTG) strategy highlighted discontinuity, funding cuts, frequent changes in political leadership, and other factors that contributed to failures to meet CTG targets, concluding that “[b]y 2014–15, the CTG Strategy as a coherent, national response to Indigenous disadvantage was effectively over” [44]. The most recent Australian Government report on CTG noted that, while some improvements had been achieved in addressing socioeconomic determinants of health, such as educational attainment, the gaps in Indigenous adult and child and mortality rates had not narrowed and the target to close the gap in life expectancy was not on track [3]. In recognition of this lack of progress, Australian state and federal governments have recently announced a ‘reset’ of CTG targets with increased emphasis on Indigenous community control of efforts to close the gap, albeit without additional funding [45]. Inclusion of novel health-related factors, such as the human microbiota, in the Indigenous health management armamentarium could help to further strengthen efforts to close the health gap.

### The human oral microbiota in oral and systemic disease

The human oral microbiota refers to the collection of micro-organisms living in the human oral cavity. Within this collection, there are several distinct communities of microbes that establish themselves in different habitats. The composition of these communities differs among different sites, such as the teeth, gingiva (gums), tongue, saliva, and the buccal mucosa (inside of the cheek) [46–49]. Dental plaque, a microbial biofilm that grows on the surface of teeth, has a particularly distinct composition [47, 50]. Teeth are the only surface in the body that do not regularly shed cells, allowing a complex biofilm to flourish on the tooth surface [51]. Gram-positive primary colonisers such as *Streptococcus* and *Actinomyces* species first bind directly to the acquired pellicle, a thin layer of salivary glycoproteins on the tooth surface [52]. As the plaque community grows, it can incorporate ‘bridging’ organisms, such as *Fusobacterium*, that bind to both primary and late colonisers, and Gram-negative late colonisers such as *Porphyromonas* species [52, 53]. Mature dental plaque is a highly structured community in which bacteria work together to optimise their access to appropriate resources [54]. Dental plaque, in the absence of regular oral hygiene, calcifies into a hardened calcium phosphate matrix known as dental calculus or tartar [55]. In this process, microbial DNA can be preserved in dental calculus for thousands of years, allowing for the study of microbial communities through time [56]. Despite these useful qualities of dental plaque and calculus communities, lower invasiveness and ease of collection has favoured the use of saliva as a proxy for studying the oral microbiota as a whole in modern-day populations. Although saliva may not represent a true microbial ‘community’, it can provide broad insight into the microbes present throughout the oral cavity [47, 50].

Oral microbial communities have been implicated in the most common oral diseases: dental caries, periodontal disease, and oral cancers [57]. Dental caries affects approximately 90% of the global population and can be highly resistant to interventions [57–59]. This disease is primarily caused by oral bacteria fermenting sugars to acid, which in turn dissolves the enamel microstructures in the outer layer of the tooth to enable bacterial entry deeper into the tooth structure [60]. *Streptococcus mutans* and related oral bacteria (‘mutans streptococci’) have traditionally been considered the microorganisms responsible for caries [61–63]. However, this paradigm has been challenged by culture-independent research demonstrating that mutans streptococci can be detected in the oral microbiota of caries-free individuals and identifying alternative bacterial species associated with caries [46, 64]. Hence, the oral health field has witnessed an increasing focus on the overall ecology of the oral microbiota in relation to caries, in particular the balance between acid-producing, acid-tolerant and alkali-producing species and metabolic processes [65–68].

Periodontal disease refers to a group of conditions characterised by inflammation of the gingiva (gums), which can range from relatively mild and reversible gingivitis to severe periodontitis involving tissue destruction and loss of bone and teeth [69]. This inflammation is thought to constitute an immune reaction by the human host against resident oral bacteria. Although periodontitis is often characterised as a “polymicrobial infection” [70], a mixture of microbial, host, and environmental factors are likely required to produce the disease [71, 72]. Identification of specific pathogenic species in periodontal disease [73, 74], such as the ‘red complex’ bacteria, has been complicated by culture-free microbiota studies, which have both expanded the list of putative periodontal pathogens [75–77] and identified changes in oral microbiota community structure, ecological diversity, and the relative abundance of specific microbial taxa in periodontal disease compared to periodontal health [78–81]. These findings have formed the basis for new theories regarding periodontal disease, including the concepts of the ‘pathobiont’—a species that is normally harmless but has the potential to cause disease in a changed environment—and the ‘keystone pathogen’—a species that can cause disease, even when present in low abundance, through its interactions with other microbes and with the host immune system [82, 83].

Recent research also supports a role for the oral microbiota in oral cancers. Several studies have found links between periodontal disease and increased risk of oral cancer [25, 26, 84]. These links may be mediated by the production of carcinogenic compounds by the oral microbiota, direct carcinogenic effects of specific microbes (particularly viruses), or the chronic inflammation resulting from periodontal disease [25, 85]. Although the role of the oral microbiota in oral cancer is a relatively new field of study, differences in the relative abundance of specific bacterial taxa and the presence of unique taxa in oral cancer compared to clinically healthy sites have been reported [86, 87], suggesting that the topic warrants further attention. For all three of these diseases, a deeper understanding of the role of the oral microbiota is needed to improve our ability to treat and prevent these conditions, especially in groups, such as Indigenous Australians, who disproportionately experience poor oral health.

In addition to its roles in widespread oral diseases, the oral microbiota has direct and indirect impacts on many systemic diseases. On the one hand, there is considerable evidence to support a role for specific oral species in the initiation or progression of certain non-oral conditions. A classic example is infective endocarditis, a life-threatening illness frequently triggered by oral *Streptococcus* species [28, 88]. Although these species typically live harmlessly in the oral cavity, they can cause severe disease when they escape into the bloodstream (termed bacteraemia) and colonise injured heart endothelial tissue [88]. Another common oral species, *Fusobacterium nucleatum*, is frequently isolated from colorectal tumours and is thought to promote colorectal cancer through several mechanisms, including promotion of a pro-inflammatory host response (reviewed in [89]) and tumour microenvironment [90], acceleration of cancer cell proliferation through activation of Wnt/beta-catenin signalling [91], interference with the host immune response [92, 93] and induction of chemotherapy resistance [94]. It has recently been shown that *F. nucleatum* strains from patient colorectal tumours are also found in saliva from the same individual, and most likely reach tumours by escape from the mouth into the bloodstream [95, 96]. A third example is the emerging evidence of a possible relationship between *Porphyromonas gingivalis*, an oral species associated with periodontal disease, and Alzehimer’s disease (reviewed in [97]). *P. gingivalis* can escape from the mouth into the blood and colonise other tissues, while *P. gingivalis* products have been identified in brain autopsy specimens of humans and mice with Alzheimer’s disease. Therefore, while considerable research is needed to identify specific oral bacterial species and fully elucidate mechanisms linking them to various systemic conditions, current evidence suggests that oral bacteria may play an important role in several diseases.

More generally, the association of periodontal disease with many chronic systemic conditions suggests that the oral microbiota as a whole may exert a substantial influence on overall health [98–100]. Although the mechanisms that underlie these links are not fully understood, some hypotheses exist. Firstly, the observed relationship between periodontal and systemic diseases could be based on shared risk factors, such as smoking [20, 101]. Secondly, periodontal inflammation and bleeding could facilitate bacteraemia caused by oral bacteria which, in turn, contributes to the initiation and maintenance of systemic disease, as in the examples discussed in the previous paragraph [100, 102]. Thirdly, the long-term inflammation stimulated by periodontal disease in the mouth could affect systemic immune function, contributing to disease [16, 19, 100, 102–104]. For instance, recent evidence suggests that oral microbiota in periodontal disease may inhibit normal macrophage polarisation and function [105]. Finally, a combination of these mechanisms could also act in tandem to promote disease. A summary of representative recent publications on proposed links between the oral microbiota and non-oral or systemic NCDs is given in Table 1.

**Table 1:** Recent publications on direct and indirect links between oral microbiota and systemic NCDs

Publication	Non-oral disease(s) linked to oral microbiota	Proposed mechanism(s)
*Infective endocarditis*		
Kawamata et al. [27] [original research]	Infective endocarditis	Bacteraemia
Cahill and Prendergast [88] [review]	Infective endocarditis	Bacteraemia, colonisation of thrombus following endothelial injury
*Colorectal cancer*		
Flemer et al. [141] [original research]	Colorectal cancer	Colonisation of colorectal tumour, shared risk factors, possibly Western diet
Komiya et al. [95] [original research]	Colorectal cancer	Colonisation of colorectal tumour from origins in the mouth
Abed et al. [96] [original research]	Colorectal cancer	Bacteraemia, colonisation of colorectal tumour via bloodstream
*Central nervous system diseases*		
Ryder [97] [review]	Alzheimer’s disease	Bacteraemia, infiltration of central nervous system, gingipain activity
Ballini et al. [105] [original research]	Central nervous system diseases	Perturbed host tissue homeostasis and immune response, chronic host inflammatory response, carriage of pathogens between body sites by macrophages
*Cardiovascular disease*		
Lockhart et al. [101] [review]	Cardiovascular disease	Shared risk factors, systemic host inflammatory response
Schenkein and Loos [104] [review]	Cardiovascular disease	Systemic host inflammatory response, cross-reactive antibody production, promotion of dyslipidaemia, shared genetic susceptibility factors
Bansal et al. [102] [review]	Cardiovascular disease	Host inflammatory response, direct induction of inflammatory mediators by bacteria or bacterial products
Carrizales-Sepùlveda et al. [19] [review]	Cardiovascular disease	Systemic host inflammatory response, oxidative stress, cross-reactive autoantibody production, shared risk factors
*Diabetes mellitus*		
Lalla and Papapanou [16] [review]	Diabetes	Systemic host inflammatory response, increased proinflammatory cytokine production
*Rheumatoid arthritis*		
Horz and Conrads [142] [review]	Rheumatoid arthritis	Host immune response
Bingham and Moni [22] [review]	Rheumatoid arthritis	*Porphyromonas gingivalis* enzyme activity, host inflammatory response
*Mutiple systemic diseases*		
Kim and Amar [103] [review]	Cardiovascular disease, diabetes, adverse pregnancy outcomes, osteoporosis	Direct invasion of host tissues, systemic host inflammatory response, changes in host proinflammatory cytokine and hormone levels, shared risk factors
Borgnakke [98] [review]	Cardiovascular disease, diabetes, metabolic syndrome, non-alcoholic fatty liver disease, rheumatoid arthritis, chronic kidney disease	Bacteraemia, microbial dysbiosis, host inflammatory response, indirect stimulation of immune response by bacterial products
Guimarães et al. [99] [book chapter]	Diabetes, cardiovascular disease	Bacteraemia, host inflammatory response
Joshipura and Andriankaja 2016 [143] [book chapter]	Cardiometabolic conditions	Common risk factors, host inflammatory response

Representative articles and book chapters published since 2005 are listed, along with the non-oral disease(s) and proposed mechanistic links with the oral microbiota discussed in each publication

### The oral microbiota of Indigenous peoples

The many host-related and environmental factors that shape the composition and activity of the oral microbiota represent an area of active research. Microbial community composition at different oral sites is influenced by local ecological factors such as nutrient and oxygen availability, pH, and salivary flow rate [106, 107]. The oral microbiota appears to be at least partly vertically transmitted from parent to child, although the relative importance of inherited host genetic factors versus parent-to-child transfer of microbes through physical contact and shared environment is debated [108–115]. A small number of studies have reported correlations between specific dietary nutrients and oral microbiota composition and diversity in modern humans, while major shifts in the oral microbiota composition of ancient Europeans have been attributed to dietary changes following the introduction of agriculture [116–118]. Current evidence suggests that the oral microbiota is resilient to the effects of short-term antibiotic use, but clinical reports indicate that long-term, heavy antibiotic use can damage endogenous oral bacterial communities, in addition to having systemic impacts on the gut and inflammation [60, 119]. Therefore, current evidence indicates that the oral microbiota is influenced by a combination of vertical transmission, host genetics, and lifestyle-related factors.

This evidence raises the pertinent question of whether the oral microbiota differs among human populations with different histories and cultures. Most oral microbiota research is conducted among people of predominantly European descent living industrialised lifestyles [120, 121]. However, reconstructions of ancient oral microbiota have identified shifts in microbiota composition that may be linked to major changes in lifestyle, such as meat consumption and transition to agriculture [116, 122]. In three studies of the oral microbiota of modern-day Indigenous populations living hunter-gatherer lifestyles in Venezuela, the Philippines, and Uganda, saliva samples from Indigenous individuals exhibited a microbial composition distinct from that of industrialised or farming populations [123–125]. A fourth study found that Cheyenne and Arapaho Native Americans residing in towns in western Oklahoma had much higher variability in oral microbiota composition than did non-Native individuals living nearby [120]. All four of these studies established differences in within-individual diversity (alpha diversity) and inter-individual variability (beta diversity) of the oral microbiota when comparing Indigenous to non-Indigenous individuals [120, 123–125]. Preliminary work in Australia shows that the oral microbiota of Indigenous Australians living industrialised lifestyles differ significantly from those of non-Indigenous Australians [126]. Although current knowledge is not extensive and suffers from some limitations, it seems credible that Indigenous individuals may harbour oral microbiota that differ in important ways from those of non-Indigenous individuals, even in a shared context of industrialisation.

A number of factors could plausibly influence the Indigenous Australian oral microbiota in unique ways. For example, smoking is more common among Indigenous Australians than in the overall Australian population, and has previously been shown to influence the composition of the oral microbiota [79, 127]. Diet may also influence the oral microbiota and is likely to vary among Indigenous Australians compared to non-Indigenous Australians. For instance, health survey data has reported that many Indigenous Australians obtain a comparatively large proportion (on average, 41%, compared to 35% for the overall Australian population) of their dietary energy from ‘discretionary’ foods such as confectionery, snack foods and alcohol [4, 128]. Reported consumption of free sugars is relatively high among Indigenous Australians [4], which may impact the oral microbiota by, for example, favouring the growth of sugar-metabolising bacteria. Traditional foods such as native game meats, certain insects, and native plants are rarely eaten by non-Indigenous Australians and may impart a unique effect on the oral microbiota, whether through the introduction of different environmental microbes or through other nutrients or compounds that could affect resident microbial communities.

Finally, the evolutionary history of the microbiota in Australia may explain some differences between the oral microbiota of Indigenous and non-Indigenous Australians. Archaeological and genetic evidence suggests that Aboriginal Australians had lived on Country (i.e. in specific homelands), in relative isolation from the rest of the world, for at least 50,000 years prior to European arrival [129, 130]. Under this scenario, we might expect that the microbiota of Indigenous Australians became uniquely adapted to the Australian environment and to aspects of human lifestyles, such as diet and medicines. Alternatively, even without specific adaptation, a process of random microbiota ‘drift’ as people settled in specific homelands may have led to variation in microbiota across the continent. As current evidence suggests that the oral microbiota is partly heritable, such historic variation in the microbiota could explain differences between the oral microbiota of Indigenous and non-Indigenous Australians, even when living relatively similar industrialised lifestyles today. Given the links between the oral microbiota and oral and systemic disease, such differences could have important implications for Indigenous health in terms of both disease risk and response to treatments. Indeed, Skelly et al. [121] have previously argued that the traumatic experience of colonisation and its possible impacts on the human microbiome are likely to have health consequences for Indigenous people today.

### Harnessing oral microbiota research to benefit Indigenous communities

There are numerous potential benefits from investigating the oral microbiota in Indigenous Australians. As reviewed above, a significant body of evidence points towards a central role for the oral microbiota in shaping oral and overall health. The ability to understand and shape the microbiome to support human health holds considerable promise for addressing the burden of microbiome-mediated diseases, perhaps especially for groups whose access to medical care is compromised by structural issues such as geographic remoteness. Novel microbiome-based therapies, such as oral microbiota transplants [131, 132] and probiotic mouthwash or lozenges [133–138], are of increasing interest in the field of oral health promotion. However, factors including poor historical and continuing research practices when engaging with Indigenous communities have led to a dearth of studies examining how the human microbiome mediates disease in Indigenous populations [139]. Therefore, there is currently little understanding of how existing treatments may influence the microbiota of Indigenous populations and few attempts to verify that microbiome-based therapies will be effective for these groups. Explicit inclusion of Indigenous Australians in oral microbiota research could help strengthen efforts to close the Indigenous health gap and to ensure that future health benefits stemming from microbiota research in general are equitably shared. To realise this goal, it will be crucial to continue to strive for ethical and culturally appropriate conduct of research, including commitment to the involvement and leadership of Indigenous researchers and communities in study design, data management and knowledge translation [106, 115].

Beyond these potential longer-term benefits, there are numerous opportunities for microbiota research to be a positive for Indigenous communities in the short term. Importantly, it may be some time before robust and sufficiently powered microbiota research is carried out, analysed, and translated into new therapies or healthcare approaches with tangible benefits for Indigenous communities [121]. Valid concerns have previously been raised about the dangers of over-promising therapeutic benefits of microbiota research to research participants, especially for marginalised groups such as Indigenous peoples [140]. However, research could be designed in such a way as to provide other, non-medical benefits for Indigenous Australians in the shorter term. Oral microbiota research could present opportunities for collaborative research partnerships between scientists and Indigenous communities, for two-way outreach and knowledge-sharing, and for scientific training and capacity-building among Indigenous Australian communities through programs such as the Summer Internship for Indigenous Peoples in Genomics (SING) Australia.

## Conclusion

Chronic systemic NCDs such as cardiovascular disease, diabetes, chronic kidney disease, and cancer disproportionately impact Indigenous Australians and have known associations with poor oral health. The human oral microbiota is linked to the pathogenesis of oral diseases that severely affect Indigenous Australians. Current evidence suggests that oral microbiota composition and diversity differ between Indigenous and non-Indigenous people in Australia and elsewhere. Therefore, the oral microbiota and oral health are likely to be directly linked to oral and systemic health outcomes in Indigenous peoples. Appropriate inclusion of Indigenous Australians in oral microbiota research and further investigation of these links between the oral microbiota and oral and systemic disease is an important emerging strategy to help close the Indigenous health gap in Australia.

## Data Availability

Data sharing is not applicable as no datasets were generated or analysed during the current study.

## Abbreviations

CTG: Closing the gap
NCD: Non-communicable disease
NSAOH: National Survey of Adult Oral Health
